# Tryptophan metabolites profile predict remission with dietary therapy in pediatric Crohn’s disease

**DOI:** 10.1177/17562848251323004

**Published:** 2025-02-25

**Authors:** Rotem Sigall Boneh, Nikki van der Kruk, Eytan Wine, Charlotte M. Verburgt, Tim G. J. de Meij, Mark Löwenberg, Krisztina B. Gecse, Nicolette Wierdsma, Joep P. M. Derikx, Wouter J. de Jonge, Geert D’Haens, Mohammed Ghiboub, Johan E. Van Limbergen

**Affiliations:** Department of Pediatric Gastroenterology and Nutrition, Emma Children’s Hospital, Amsterdam University Medical Centers, Amsterdam University, Amsterdam, The Netherlands; Tytgat Institute for Liver and Intestinal Research, Amsterdam Gastroenterology Endocrinology Metabolism, Amsterdam University Medical Centers, Amsterdam University, Amsterdam, The Netherlands; Pediatric Gastroenterology and Nutrition Unit, The E. Wolfson Medical Center, Holon, Israel; Department of Pediatric Gastroenterology and Nutrition, Emma Children’s Hospital, Amsterdam University Medical Centers, Amsterdam University, Amsterdam, The Netherlands; Tytgat Institute for Liver and Intestinal Research, Amsterdam Gastroenterology Endocrinology Metabolism, Amsterdam University Medical Centers, Amsterdam University, Amsterdam, The Netherlands; Division of Pediatric Gastroenterology, Department of Pediatrics, University of Alberta, Edmonton, AB, Canada; Department of Pediatric Gastroenterology and Nutrition, Emma Children’s Hospital, Amsterdam University Medical Centers, Amsterdam University, Amsterdam, The Netherlands; Department of Pediatric Gastroenterology and Nutrition, Emma Children’s Hospital, Amsterdam University Medical Centers, Amsterdam University, Amsterdam, The Netherlands; Department of Gastroenterology and Hepatology, Amsterdam University Medical Centers, Amsterdam University, Amsterdam, The Netherlands; Department of Gastroenterology and Hepatology, Amsterdam University Medical Centers, Amsterdam University, Amsterdam, The Netherlands; Department of Gastroenterology and Hepatology, Amsterdam University Medical Centers, Amsterdam University, Amsterdam, The Netherlands; Department of Pediatric Surgery, Emma Children’s Hospital, Amsterdam University Medical Centers, Amsterdam University, Amsterdam, The Netherlands; Tytgat Institute for Liver and Intestinal Research, Amsterdam Gastroenterology Endocrinology Metabolism, Amsterdam University Medical Centers, Amsterdam University, Amsterdam, The Netherlands; Department of Gastroenterology and Hepatology, Amsterdam University Medical Centers, Amsterdam University, Amsterdam, The Netherlands; Department of Surgery, University of Bonn, Bonn, Germany; Department of Gastroenterology and Hepatology, Amsterdam University Medical Centers, Amsterdam University, Amsterdam, The Netherlands; Tytgat Institute for Liver and Intestinal Research, Amsterdam Gastroenterology Endocrinology Metabolism, Amsterdam University Medical Centers, Amsterdam University, Meibergdreef 69, Amsterdam 1105 BK, The Netherlands; Department of Pediatric Surgery, Emma Children’s Hospital, Amsterdam University Medical Centers, Amsterdam University, Amsterdam, The Netherlands; Tytgat Institute for Liver and Intestinal Research, Amsterdam Gastroenterology Endocrinology Metabolism, Amsterdam University Medical Centers, Amsterdam University, Meibergdreef 9, Amsterdam 1105 AZ, The Netherlands; Department of Pediatric Gastroenterology and Nutrition, Emma Children’s Hospital, Amsterdam University Medical Centers, Amsterdam University, Amsterdam, The Netherlands; Amsterdam Public Health Research Institute, University of Amsterdam, Amsterdam, The Netherlands

**Keywords:** dietary therapy, feces, pediatric Crohn’s disease, prediction of remission, tryptophan metabolites

## Abstract

**Background:**

Crohn’s disease (CD) exclusion diet combined with partial enteral nutrition (CDED + PEN) or exclusive enteral nutrition (EEN) is effective in inducing remission in mild-to-moderate pediatric CD. Although CDED + PEN is better tolerated and has higher compliance compared to EEN, a subset of patients does not achieve remission. Diet-induced remission is shown to be positively associated with specific changes in tryptophan-metabolites.

**Objectives:**

To investigate whether the abundance of baseline fecal tryptophan-metabolites predicts dietary therapy outcomes in pediatric CD.

**Design:**

Diagnostic accuracy study and secondary analysis of previously conducted Randomized Controlled Trial (RCT).

**Methods:**

Twenty-six patients from previously performed RCT of mild-to-moderate pediatric CD were included. The patients were classified as having clinical remission (R) (*n* = 19 in total; CDED + PEN = 10 and to EEN = 9) or No-Remission (NR) (*n* = 7 in total; CDED + PEN = 3 and EEN = 4) following 6 weeks of therapy, based on the Pediatric Crohn’s Disease Activity Index score (⩽10 = remission). We performed a targeted quantitative analysis of 21 tryptophan-metabolites in baseline (*t* = 0) fecal samples from both groups, utilizing liquid chromatography coupled with quadrupole mass spectrometry. Receiver operator characteristic curve (ROC) and random forest analysis (RFA) were used to assess the predictive power of fecal tryptophan-metabolites for dietary outcomes at baseline. Ratios of tryptophan-metabolites were compared to investigate different downstream tryptophan pathways.

**Results:**

Baseline fecal kynurenine level was significantly higher in NR compared to R for CDED + PEN (*p* = 0.02) and EEN (*p* = 0.04). ROC analysis highlighted the robust predictive power of kynurenine for CDED + PEN (area under the curve (AUC = 0.97)) and EEN (AUC = 0.88)-induced remission. RFA corroborated these observations. The ratio serotonin/kynurenine was the strongest predictor of CDED + PEN-induced remission (AUC = 1). The ratio 5-hydroxytryptophan/kynurenine (AUC = 0.88) predicted EEN-induced remission. By combining data from CDED + PEN and EEN, kynurenine (AUC = 0.91) and ratios of quinolinic acid/kynurenine (AUC = 0.93) and kynurenine/indole-3-acetic acid (AUC = 0.88) demonstrated strong predictive performance for dietary therapy-induced remission.

**Conclusion:**

Baseline tryptophan metabolites have the potential to serve as a biomarker for dietary remission in pediatric CD. Some tryptophan metabolite ratios showed the most promising predictive capabilities. If confirmed in validation studies, baseline fecal tryptophan markers may be able to provide much-needed guidance to personalize dietary intervention within the management of pediatric CD.

**Trial registration:**

NCT01728870.

## Introduction

The increasing incidence of pediatric Crohn’s disease (CD), a type of inflammatory bowel disease (IBD), has been strongly associated with a shift toward the Westernized diet.^1–3^ While the etiology of CD remains largely unclear, multifactorial, and mostly complex polygenic, multiple studies have underscored the pivotal role of the gut microbiome and its interplay with dietary factors in the disease’s pathogenesis.^4,5^

The Westernized diet, characterized by a high intake of processed foods, refined sugars, saturated fats, and additives, has been linked to increased inflammation, altered gut microbial communities, and metabolic profiles including tryptophan.^6–9^ Together, these lifestyle factors have been shown to contribute to both the onset and progression of CD.^10–14^ Although dietary guidance is identified as a key priority by patients and patient organizations, it is often not offered as part of standard IBD management.^15–18^

Several recent ECCO, ECCO/ESPGHAN, and ESPEN guidelines and reports have raised the profile of dietary therapy, for mild-to-moderate disease or as add-on therapy, for example, when additional immune suppression is undesirable.^9,19–25^ Meta-analyses have confirmed the superiority of nutritional therapy over corticosteroids for induction of clinical remission and mucosal healing using exclusive enteral nutrition (EEN), as well as better postoperative outcomes when used pre-operatively.^26–28^

The Crohn’s disease exclusion diet (CDED) combined with partial enteral nutrition (PEN) as well as EEN are both effective at inducing remission in mild-to-moderate CD but CDED + PEN is better tolerated.^29–31^ Both dietary options aim to reduce inflammatory dietary triggers while providing essential nutrients, but for most patients, the anti-inflammatory effect is quickly lost when normal diet is resumed.^29,30,32,33^ While both dietary therapies have shown great effectiveness in inducing remission and improving clinical outcomes, adherence can be challenging. In addition, one-third of patients do not respond to dietary therapy.^30,31,34,35^

Tryptophan, an essential amino acid primarily derived from dietary intake, plays a central role in several physiological functions.^36^ Tryptophan is a precursor for the synthesis of various bioactive molecules, including serotonin, indoles, kynurenine, and serotonin metabolism pathway components.^37^ Recent studies have emphasized the importance of tryptophan metabolic pathways in modulating intestinal immunity and maintaining gut homeostasis through activation of the AhR.^38–41^

In the intestine, tryptophan is metabolized along three main routes, leading to kynurenine, serotonin, and indole derivative synthesis, under the direct or indirect involvement of the microbiota.^42,43^ Alterations in gut microbiome composition, associated with CD, can consequently affect tryptophan.^36,43,44^ Our group and others have shown the association of tryptophan metabolism with intestinal and systemic inflammation, both when measured in serum and in feces, as well as using two-sample Mendelian randomization of available genome-wide association studies datasets.^39,42,45,46^ Harris et al.^45^ have recently described reduced serum tryptophan levels across the majority of chronic inflammatory diseases (as evident by an inverse relationship between tryptophan and C-reactive protein (CRP). It is likely that increases in the kynurenine-to-tryptophan ratio (kynurenine/tryptophan)^45^ reflect the tryptophan metabolic pathway in inflammation through the enzymatic activity of indoleamine 2,3-dioxygenase (IDO)/tryptophan 2,3-dioxygenase in myeloid reactive immune cells.

In turn, achieving clinical remission of CD alters tryptophan metabolism, both with immune suppression and dietary therapy.^36,47^ Wolf et al.^47^ previously showed that the tryptophan-metabolizing enzyme IDO expression, which in turn affects T-cell proliferation and survival, is reduced by anti-TNF therapy. In a translational research report of the index CDED-Randomized Controlled Trial (RCT), we showed that the strong reduction in kynurenine level was associated with induced and sustained remission and explained the association with the kynurenine/tryptophan ratio.^36^ A subsequent expanded analysis of tryptophan-metabolites in the same cohort, demonstrated that changes in specific tryptophan metabolites performed well as biomarkers of nutritional therapy outcome.^36^ More specifically, a reduction in the kynurenine pathway activity and an increase in serotonin pathway activity was associated with successful induction of, as well as sustained, remission to both CDED + PEN and EEN.^42^

Adherence to any form of dietary therapy is the key requirement for remission which in turn can help ascertain the degree to which the presenting phenotype is “diet-responsive.”^48–51^ Given the significant lifestyle changes required for successful dietary therapy, an early indicator of likely remission is needed to personalize early treatment.^34^

In this study, we aim to analyze the tryptophan metabolite profile at baseline (pre-therapy) and investigate its potential to predict remission at Week 6 using either CDED + PEN or EEN.

## Methods

### Participants

Participants are from a previously conducted 12-week RCT.^29^ In brief, children (4–18 years) with mild to moderate luminal CD (Pediatric Crohn’s Disease Activity Index, PCDAI >10 and ⩽40) and active inflammation within 36 months of diagnosis were eligible. Exclusion criteria included recent steroid use, immunomodulator adjustments, biologics use, primary colonic disease with rectal involvement, or active perianal disease. Of the original 78 participants from the RCT,^29^ this subanalysis focused on 26 participants for whom baseline fecal samples were available for tryptophan metabolite analysis. This subset was selected due to the availability of stored samples following prior studies on metagenomics, 16S microbiome profiling, and metabolomics.^29,36,52^

### Study design

Twenty-one tryptophan metabolites were quantified in 26 fecal samples collected from 26 pediatric CD patients at baseline, before therapy initiation, during a previously conducted 12-week RCT employing two distinct nutritional therapies: CDED + PEN and EEN for inducing remission in mild-to-moderate pediatric CD conducted in Israel and Canada, with sampling at baseline and Week 6.^29^

The first group was assigned to the CDED, with additional PEN (MODULEN IBD; Nestlé Health Science, Vevey, Switzerland) accounting for 50% of their calculated energy requirements for a duration of 6 weeks. This was followed by a transition to a step-down diet involving an additional 25% of energy requirements from PEN until Week 12, as previously described.^29^ The second group exclusively followed 100% EEN for 6 weeks, after which they gradually resumed a regular diet with 25% of their caloric intake coming from PEN, as detailed in prior reports.^29^ The primary outcome measure in the RCT focused on assessing the tolerability of the dietary therapy. Secondary endpoints of the study included intention-to-treat remission at Week 6, defined by a PCDAI score of PCDAI ⩽ 10, and corticosteroid-free intention-to-treat sustained remission at Week 12. The study received ethical approval from the local ethics boards at each participating site, and informed consent was obtained from all participants (NCT01728870).

The primary objective of this study was to investigate whether any of the fecal tryptophan metabolites at baseline showed predictive capacity for therapy outcome (Remission (R) and No-Remission (NR)) after 6 weeks of dietary therapy. Thus, fecal samples that were analyzed in this study were collected before the initiation of dietary therapy (time = 0).

### Subjects

Pediatric CD patients were stratified at baseline into remission (R) and no remission (NR) groups for CDED + PEN and EEN therapy, based on achieving remission or non-remission, defined by a PCDAI score of ⩽10 or >10, respectively, at Week 6.

The distribution of CDED + PEN fecal samples at baseline was as follows (total: *n* = 13): R (*n* = 10) and NR (*n* = 3). For EEN fecal samples at baseline, the distribution was (total: *n* = 13): R (*n* = 9) and NR (*n* = 4).

Baseline characteristics and phenotype of patients are presented in Table 1. CONSORT Checklist and study flow diagram were previously published.^29^

**Table 1.** Baseline characteristics of patients.

Variable	Non-remission (patients at baseline who fail to achieve remission at week 6) (*n* = 7)	Remission (patients at baseline who achieve remission at week 6) (*n* = 19)	*p*-Value
Dietary therapy (*n*)	CDED + PEN (*n* = 3) EEN (*n* = 4)	CDED + PEN (*n* = 10) EEN (*n* = 9)	
Age, year (mean ± SD)	15.20 (±1.47)	13.73 (±1.26)	0.25
Gender (males%)	3 (42.8)	14 (73.6)	0.15
PCDAI (median, IQR)	28.93 (10–40)	26.32 (12.5–37.5)	0.97
CRP, mg/L (median, IQR)	2.45 (0.5–5.21)	3.12 (0.5–7.5)	0.58
Immunomodulators, *n* (%)	2 (28.5)	2 (10.5)	0.27
Disease location Paris classification, *n*			0.98
L1	3	6	
L2	1	0	
L3	2	12	
L4	1	1	
L4a	1	8	
L4b	2	3	
L4a + b	2	2	
Not L4	2	6	

Source: Adapted from Levine et al.^29^

CDED, Crohn’s disease exclusion diet; CRP, C-reactive protein; EEN, exclusive enteral nutrition; IQR, interquartile range; PCDAI, Pediatric Crohn’s Disease Activity Index; PEN, partial enteral nutrition.

Each patient was supplied with two containers, which were labeled with stickers for easy identification. Upon sample collection (within a 24-h window), the container holding calprotectin was stored at −20°C, while the microbiome/metabolome container was divided into four transfer tubes (Eppendorf tubes with black caps) using a wooden stick. These tubes were immediately stored at −80°C, without the addition of any buffer.

### Fecal tryptophan metabolites quantification

A targeted quantitative assessment of tryptophan metabolites in baseline fecal samples was conducted using liquid chromatography coupled with quadrupole mass spectrometry, as previously described.^53,54^

To measure fecal tryptophan metabolites, we initiated the process by combining 3 mg of lyophilized stool samples with 900 μL of a MeOH/H_2_O mixture (1:1) alongside internal standards. Subsequently, these samples were subjected to vortexing, homogenization, and centrifugation. Following this, 100 µL of the supernatants were carefully transferred to a 96-well plate for quantification of the metabolites, while the remaining 800 µL were subjected to evaporation to dryness utilizing a vacuum concentrator (Savant SPD 111 vs SpeedVac; Thermo Fisher Scientific, Waltham, USA) at 45°C over a period of 4 h. The resulting residues were then reconstituted in 100 μL of MeOH/H_2_O mixture (1:9), with 2 µL of the reconstituted solution being analyzed.

For the quantification via liquid chromatography-tandem mass spectrometry, we followed the previously established method.^53^ Calibration curves for each metabolite were established by calculating the intensity ratio between the metabolite and its corresponding internal standard. These calibration curves were subsequently employed to determine the concentrations of individual metabolites in the fecal samples. Detailed information regarding the specific calibration ranges and concentrations of internal standards, including the associated fragmentation parameters are described in Ghiboub et al.^42^

**Figure 1.**
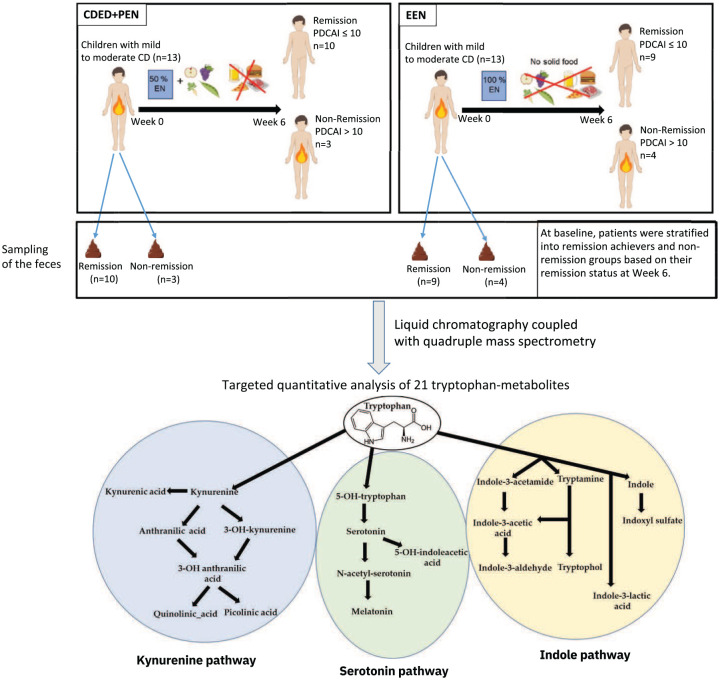
Study design depicting remission and non-remission, with fecal sample sizes at baseline. *n* = 13 for patients on EEN and *n* = 13 for patients on CDED with PEN (CDED + PEN). Fecal samples at baseline (Week 0) were collected from patients subsequently categorized as responders and non-responders based on their clinical outcome of remission or non-remission at Week 6. Remission was defined as a PCDAI score ⩽10. The fecal samples were subjected to targeted quantitative analysis of 21 tryptophan metabolites. The schematic representation illustrates the positions of all measured metabolites in various tryptophan metabolism pathways. Source: This diagram is updated from Levine et al.^29^ and Ghiboub et al.^42^ CDED, Crohn’s disease exclusion diet; EEN, exclusive enteral nutrition; PCDAI, Pediatric Crohn’s Disease Activity Index; PEN, partial enteral nutrition.

The set of 21 measured tryptophan metabolites encompassed a range of compounds, including tryptophan itself, metabolites from the kynurenine pathway (such as kynurenine, kynurenic acid, anthranilic acid, 3-OH-kynurenine, 3-OH-anthranilic acid, picolinic acid, and quinolinic acid), serotonin pathway metabolites (such as 5-OH-tryptophan, serotonin, N-acetylserotonin, melatonin, and 5-OH-indoleacetic acid), and indole pathway metabolites (including tryptamine, indoxyl sulfate (indole-3-sulfate), indole-3-acetamide, indole-3-acetic acid, indole-3-lactic acid, indole-3-aldehyde, tryptophol, and indole). Figure 1 provides a visual representation of the experimental setup and the positions of these measured metabolites within the various tryptophan metabolic pathways. The selection of these specific tryptophan metabolites was based on prior reports of their altered concentrations in various inflammatory diseases, including IBD.^36,38,42,43^

### Data processing and statistical analysis

Data preparation and subsequent statistical analyses were carried out using MetaboAnalyst 5.0, a widely recognized software web platform for metabolomics studies (https://www.metaboanalyst.ca).^55^

To enhance data quality, we employed the interquartile range method for data filtration, effectively identifying and removing variables that were deemed noisy or noninformative for subsequent modeling of the dataset, following established practices.^56^

Tryptophan metabolites were compared in three ways. We analyzed the combined data of remission (R) versus no remission (NR) for both therapies (CDED + PEN combined with EEN), as well as R versus NR specifically within the CDED + PEN and EEN groups. These comparisons allowed us to investigate potential prediction markers for dietary therapy in general, as well as for each specific dietary intervention. Receiver-operating characteristic (ROC) analysis was also conducted using MetaboAnalyst 5.0 (Xia lab, Montreal, Canada), a method previously outlined.^57^ This analysis utilized the normalized concentrations of 20 specific tryptophan metabolites. The key output measure of this analysis, the area under the ROC curve (AUC), was determined and accompanied by its corresponding 95% confidence intervals. Additionally, we instructed MetaboAnalyst 5.0 to compute and include all possible ratios among the top 20 pairs of tryptophan metabolites, based on their statistical significance (*p*-values). These ratios were subsequently incorporated into further biomarker analysis. From this comprehensive analysis, individual metabolites and metabolite ratios that exhibited an AUC of ⩾0.80 and a statistical significance level of *p* < 0.05 were singled out for further consideration. Random forest analysis (RFA) to normalized gut metabolite concentration levels with mean decrease accuracy was performed in MetaboAnalyst 5.0.^55^

The figures were adapted using Inkscape 0.92.4. For group-level analysis, the data underwent either one-way analysis of variance or Student’s *t* test, with a significance threshold set at *p* ⩽ 0.05. The data are depicted as the mean ± standard error of the mean, adhering to standard statistical reporting practices.

The reporting of this study conforms to the STARD statement.^58^

## Results

### Baseline characteristics of patients and rate of remission per group

CDED + PEN induced remission (defined as PCDAI ⩽10) in 10 (77%) out of 13 patients at Week 6 (Figure 1 and Table 1). In the EEN group, of 13 patients, 9 (69%) achieved remission at Week 6 (Figure 1 and Table 1). These numbers reflect only the patients with available fecal samples at baseline from the previously performed RCT.^29^ Similar to the RCT, analysis of this subgroup of patients revealed no significant differences in baseline characteristics (including PCDAI scores) in our subset of patients (Table 1), illustrating that the groups were well-balanced.

### Tryptophan metabolite concentrations as predictive biomarkers for non-remission to dietary intervention

**Figure 2.**
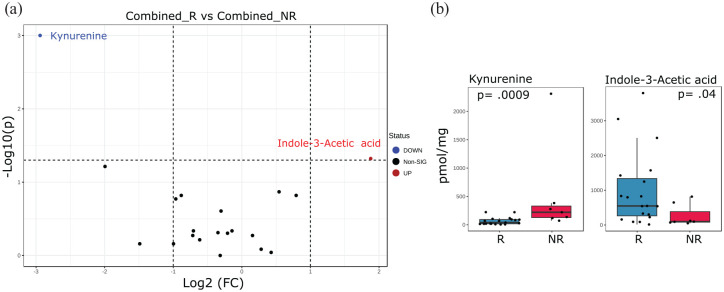
Elevated baseline fecal kynurenine and indole-3-acetic acid as predictive biomarkers for non-remission with dietary interventions (combined EEN and CDED + PEN data) in pediatric Crohn’s disease. This figure presents a combined analysis of 21 baseline fecal tryptophan metabolites, pooling data from baseline fecal samples of both EEN and CDED with PEN (CDED + PEN) therapies. The aim is to identify potential differences in fecal tryptophan metabolites at baseline that may serve as predictive biomarkers for clinical outcomes at Week 6 of dietary therapy, comparing patients who achieved R with those who did not (NR). (a) Volcano plot illustrates the differences in fecal tryptophan metabolites between the combined R and combined NR groups. Blue dots indicate significantly decreased metabolites in combined R compared to combined NR, as determined by a *t* test (*p* ⩽ 0.05). Red dots represent significantly increased metabolites, while black dots denote metabolites with no significant changes. (b) Absolute concentrations of significantly altered fecal tryptophan metabolites, specifically kynurenine and indole-3-acetic acid, comparing data from R (*n* = 19) and NR (*n* = 7) groups. Data were analyzed using a Student’s *t* test with a two-tailed significance level set at *p* ⩽ 0.05. CDED, Crohn’s disease exclusion diet; EEN, exclusive enteral nutrition; NR, no-remission; PCDAI, Pediatric Crohn’s Disease Activity Index; PEN, partial enteral nutrition; R, remission.

We analyzed potential differences in baseline fecal tryptophan metabolite concentrations between patients starting dietary therapy (combining data from both CDED + PEN and EEN groups). Of the 21 tryptophan metabolites included in the analysis, 2 exhibited significant changes (*p* ⩽ 0.05; Figure 2(a)). Baseline kynurenine level was more abundant in patients not achieving remission (*p* = 0.009), while indole-3-acetic acid was less abundant (*p* = 0.04; Figure 2(a) and (b)).

### Tryptophan metabolite concentrations as predictive biomarkers for non-remission to EEN or CDED + PEN

**Figure 3.**
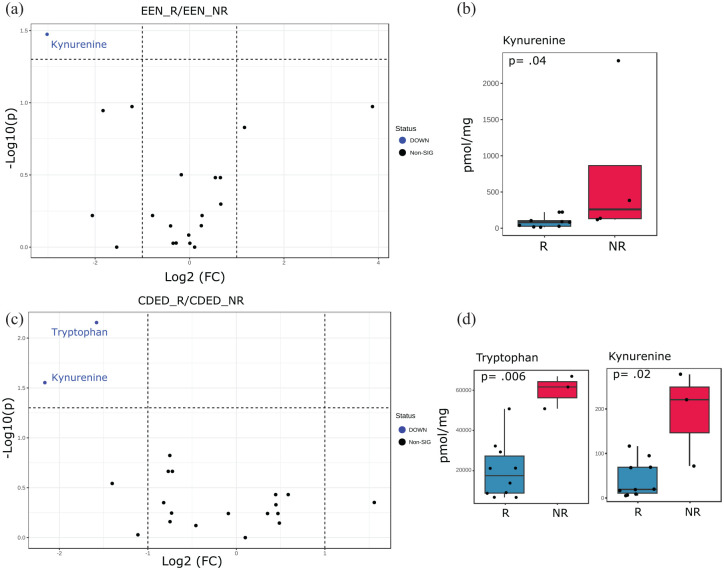
Therapy-dependent alterations in baseline fecal tryptophan metabolites predict R in pediatric Crohn’s disease. This figure presents separate analyses of 21 baseline fecal tryptophan metabolites for EEN and CDED with PEN (CDED + PEN) therapies. The aim is to identify potential differential fecal tryptophan metabolite abundances at baseline that may serve as predictive biomarkers for clinical outcomes at Week 6, specific for each dietary therapy, comparing patients who would go on to achieved R with those who would not (NR). (a) Volcano plot illustrating differences in fecal tryptophan metabolites between R and NR groups for EEN therapy. Blue dots represent significantly decreased metabolites in R fecal samples compared to NR samples (*t* test, *p* ⩽ 0.05). Black dots indicate metabolites with no significant changes. (b) Absolute concentrations of the significantly altered fecal tryptophan metabolite (kynurenine) in the EEN cohort, comparing R (*n* = 9) with NR (*n* = 4). (c) Volcano plot showing differences in fecal tryptophan metabolites between R and NR groups for CDED + PEN therapy. Blue dots represent significantly decreased metabolites in R fecal samples compared to NR samples (*t* test, *p* ⩽ 0.05). Black dots indicate metabolites with no significant changes. (d) Absolute concentrations of significantly altered fecal tryptophan metabolites in the CDED + PEN cohort, comparing R (*n* = 10) with NR (*n* = 3). Data were analyzed using Student’s *t* test with a two-tailed significance level set at *p* ⩽ 0.05. CDED, Crohn’s disease exclusion diet; EEN, exclusive enteral nutrition; NR, no-remission; PEN, partial enteral nutrition; R, remission.

To identify potential differential tryptophan metabolites abundance as predictive biomarkers specific to each type of dietary therapy, we analyzed differences in baseline fecal tryptophan metabolite concentrations between patients who would go on to achieve remission through EEN or CDED + PEN separately and those who would not. Of the 21 baseline fecal tryptophan metabolites included in the analysis, one metabolite exhibited significant changes in the EEN cohort and 2 in the CDED + PEN cohort (*p* ⩽ 0.05; Figure 3(a)–(d)). Kynurenine was significantly more abundant in fecal samples from NR patients in both EEN (*p* = 0.04) and CDED + PEN (*p* = 0.02; Figure 3(b) and (d)), while tryptophan was only significantly increased in NR fecal samples from patients on CDED + PEN (*p* = 0.006; Figure 3(a) and (c)).

### Fecal kynurenine displays high prediction signature power for non-remission

**Figure 4.**
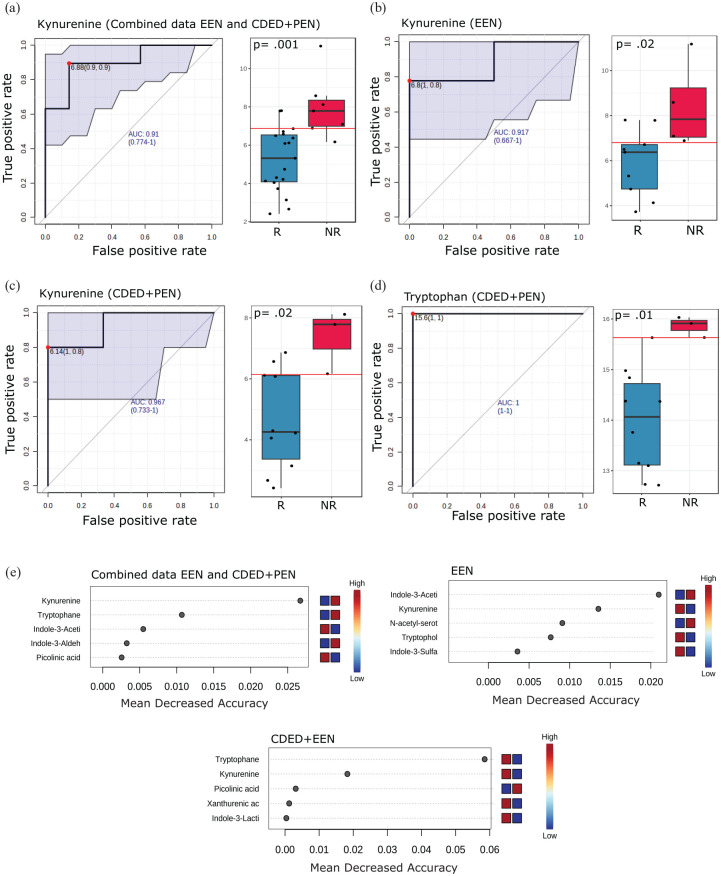
Kynurenine demonstrates high predictive power for NR with both EEN and CDED + PEN therapies. This figure presents prediction models applied to investigate the predictive power of individual fecal tryptophan metabolites at baseline to determine which patients would achieve remission at Week 6. (a–d) ROC curves with corresponding AUC scores for fecal kynurenine in (a) combined CDED + PEN and EEN data (R: *n* = 19, NR: *n* = 7), (b) EEN alone (R: *n* = 9, NR: *n* = 4), (c) CDED + PEN alone (R: *n* = 10, NR: *n* = 3), and (d) tryptophan in CDED + PEN alone. Box plots illustrate raw values with associated *p*-values. This analysis compares patients who would achieve R with those who would not (NR). (e) Random forest analysis of normalized fecal tryptophan metabolite concentration levels. The top 5 metabolites with the highest discriminatory power between R and NR are shown for combined CDED + PEN and EEN data, EEN alone, and CDED + PEN alone. Red fields indicate high abundance (concentration), and blue fields indicate low abundance of the particular metabolite based on conditions. AUC, area under the ROC curve; CDED, Crohn’s disease exclusion diet; EEN, exclusive enteral nutrition; NR, no-remission; PEN, partial enteral nutrition; R, remission; ROC, receiver operating characteristic.

ROC analysis showed kynurenine as the only tryptophan metabolite with a strong discriminatory power to predict the NR when CDED + PEN and EEN data are combined or for EEN alone (AUC = 0.91, *p* = 0.001 and AUC = 0.91, *p* = 0.02, respectively; Figure 4(a) and (b)). For CDED + PEN, kynurenine was the second strongest predictive biomarker with AUC = 0.96 and *p* = 0.02, after tryptophan with AUC = 1 and *p* = 0.01 (Figure 4(c) and (d)). Kynurenine was also one of the most important metabolites as a predictor in the RFA, corroborating the data generated from ROC analysis (Figure 4(e)).

### Tryptophan metabolite ratios as a potential biomarker to predict non-remission

**Figure 5.**
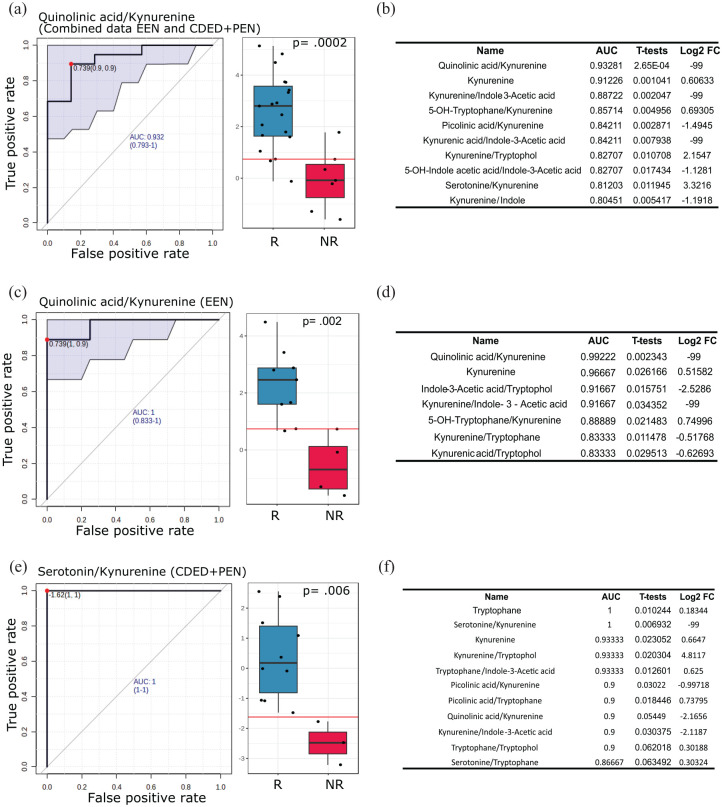
Tryptophan metabolite ratios as potential biomarkers for predicting remission in dietary interventions for pediatric Crohn’s disease. This figure presents ROC analyses of fecal tryptophan metabolite ratios to evaluate their predictive power for remission at Week 6 following dietary interventions. This analysis compares fecal samples from patients who achieved R versus those who did not (NR). (a, c, and e) ROC curves with corresponding AUC scores for the most significant metabolite ratios (highest AUC score) in (a) combined EEN and CDED + PEN data (R: *n* = 19, NR: *n* = 7), (c) EEN alone (R: *n* = 9, NR: *n* = 4), and (e) CDED + PEN alone (R: *n* = 10, NR: *n* = 3). Box plots display raw values with associated *p*-values. (b, d, and f) Summary tables of the most predictive signatures based on ROC analysis for individual metabolites and metabolite ratios. Ratios with AUC > 0.8 and *p* ⩽ 0.05 were considered significant for (b) combined EEN and CDED + PEN data, (d) EEN alone, and (f) CDED + PEN alone. In the tables, negative Log2 FC values indicate lower ratios in NR compared to R, while positive values indicate higher ratios in NR compared to R. AUC, area under the curve; CDED, Crohn’s disease exclusion diet; EEN, exclusive enteral nutrition; FC, fold change; NR, no-remission; PEN, partial enteral nutrition; R, remission; ROC, receiver operating characteristic.

Metabolism is a dynamic process where catabolism and anabolism occur continuously, thus ratios between metabolites within the same human subject may yield better biomarkers and provide better overview about the specific pathway metabolism activity. Therefore, we opted to perform ROC analysis computed on metabolite ratios (pairwise). By applying the cut-off of AUC > 0.8 and *p* ⩽ 0.05, we detected 9, 6, and 9 tryptophan metabolite ratios as prediction signatures for clinical outcome of the combined data of CDED + PEN and EEN, EEN alone, and CDED + PEN alone, respectively (Figure 5).

In general, the ratio of quinolinic acid-to-kynurenine displayed the most promising prediction signature of remission when combining the data of CDED + PEN and EEN or EEN alone, with AUC = 0.93, *p* = 0.002 and AUC = 1, *p* = 0.02, respectively (Figure 5(a)–(d)). While the ratio of serotonin-to-kynurenine (AUC = 1 and *p* = 0.006; Figure 5(e) and (f)) was ranked as the best predictor for CDED + PEN therapy remission, quinolinic acid-to-kynurenine also displayed a high prediction power with AUC = 0.9 (Figure 5(f)). It is worth mentioning that the ratio of kynurenine-to-indole-3-acetic acid displayed high prediction power for both nutritional therapies, as well as when we combined their data, with AUC > 0.8 and *p* ⩽ 0.05. Some other interesting ratio signatures are also listed in Figure 5(b), (d), and (f).

## Discussion

In this study, we have shown that tryptophan metabolites, particularly kynurenine, may have potential as biomarkers of remission with nutritional therapy in children with de novo CD. Previously, we showed the association of kynurenine and the ratio of kynurenine/tryptophan with clinical remission at Week 6 and sustained remission at Week 12.^36,42^ Herein, we show that already baseline assessment of kynurenine and ratios of tryptophan metabolites, reflecting the various downstream pathways away from kynurenine, have the potential to predict dietary remission and non-remission 6 weeks later.

For this study, we used available samples from the index RCT of CDED + PEN versus EEN which was a cohort of mostly treatment-naïve teenagers with ileal involvement.^29,48^ Previous analyses have focused on various metagenome, inferred metabolic phenotype, and unbiased metabolome analyses.^29,36,52^ We showed that the metabolic phenotype (metabotype) shifts toward healthy controls which each additional week of nutritional therapy.^52^ Fecal short chain fatty acids (SCFAs) and standard primary and secondary bile acids did not appear to be the main mechanisms of action of nutritional therapy. Instead, a wide range of other metabolic pathways including microbial amino acid metabolism were identified to play a role in achieving remission.^36^

Tryptophan metabolism has received increasing research attention in immune-mediated disease as it integrates environmental exposures including diet with inflammation in a variety of tissues, settings, and diseases. The microbiome-host interaction critical to the development and disease course in IBD influences the selective metabolism of tryptophan into the three established downstream pathways (Kynurenine, Serotonin (both host), and indole (microbial)).^42,59^ Long-term dietary patterns have repeatedly been associated with CD susceptibility and microbiome features, associated with inflammation.^14,60^ Recently, the Gene-Environment-Microbiome cohort study showed that microbiome changes predate the onset of inflammation in a cohort of siblings of patients with IBD, some of whom have gone on to develop IBD.^61,62^ Modification of dietary exposure will therefore be receiving increasing research interest in the pre-clinical/at-risk phase before the onset of inflammation.^63–65^

In our previous study,^42^ we observed that specific tryptophan metabolites, particularly those in the kynurenine pathway, such as increased levels of kynurenine and quinolinic acid, were strongly associated with non-remission to both EEN and CDED + PEN at Week 6. This finding suggests that achieving remission at Week 6 may require lower levels of these metabolites. Interestingly, our current study shows that patients who did not respond to dietary therapy at Week 6 already exhibited elevated levels of kynurenine at baseline (Week 0). This indicates that this metabolite could serve as a strong biomarker for predicting therapy outcomes, notably dietary therapy in CD. Additionally, this opens new research avenues, as this metabolite might contribute to the lack of response in some patients and could potentially be targeted to improve remission rates. The persistent elevation of these metabolites in non-responders highlights their possible role in the disease’s pathophysiology and suggests that modulation of the kynurenine pathway could be a therapeutic target. Further research is warranted to explore causal relationships and to develop targeted interventions that may enhance remission rates by addressing these metabolic pathways.

In the early stages of CD therapy, the identification of a diet-responsive phenotype can inform decisions regarding the appropriate vaccination strategy versus the timely introduction of immunomodulators/anti-TNF.^34,66^ Recently, the regulatory effect of nutritional therapy on inflammation has been described.^67–69^ In keeping with this research, restricting a dietary intervention to 6 weeks has repeatedly been shown to lead to undertreatment/early flare, particularly in the moderate-to-severe group of patients.^68^ In the landmark study by Jongsma et al.,^68,70^ azathioprine was unable to control this rebound inflammation (replicating the larger experience with thiopurines in adult CD).^19,71^ On the other hand, an indolent course of CD may benefit from and be sufficiently treated by controlling inflammatory triggers through dietary management.^30,72^

In mild-to-moderate CD (B1 in the Montreal/Paris classification), many practitioners choose to avoid corticosteroid exposure, but the best type of nutritional intervention spanning induction and maintenance of remission is still the subject of debate.^9,15,19^ A reliable marker to give an early indication of the likelihood of dietary remission has been lacking. Nichols et al.^73^ have recently identified a range of microbial signals and diet-related metabolites in feces. Paradoxically, remission associated with EEN as well as the prediction of the likelihood of remission are linked to lower levels of SCFAs. In their study, as well as in the recent larger analysis of the Canadian pediatric IBD inception cohort, pre-existing diet may be a large confounder and determinant of baseline microbiome and remission with nutritional therapy.^74^ A better understanding of pre-diagnosis diet and baseline microbiome can avoid undertreatment due to incorrect guidance. In this regard, important lessons can be drawn from previous PEN’s studies showing a significantly lower remission rate compared to EEN.^4,75,76^

Through the identification and exclusion of potential food-derived triggers of inflammation, far beyond the often selectively quoted reduction of emulsifiers, the CDED + PEN intervention has proven to be an important incremental change to the role of diet in induction and maintenance of remission extending beyond pediatric practice.^19,21,32^ Early identification of markers of the likelihood of successful induction of remission can help guide induction as well as potential for dietary maintenance.

In the search for predictive markers, it is important to discuss the characteristics of the cohort used to develop the biomarker.^77^ In the index RCT of CDED + PEN, all included patients had mild-to-moderate disease as defined by the PCDAI, were required to have ileal involvement, and not to have severe disease in the rectosigmoid.^29^ Indeed, some historical case series had suggested isolated colonic disease is less amenable to dietary treatment but lack of differentiation between right-sided colonic and distal colonic disease involvement, may be important to fully characterize the response of colonic-predominant disease.^78,79^ In the cohort included in the CDED + PEN RCT, the Paris-location of disease was not a significant predictor of remission.^48^

Dhaliwal et al.^80^ have shown a poor correlation between PCDAI and markers of endoscopic severity in pediatric CD. Also in terms of prediction of disease course, it is pertinent to note that, for example, colonic ulceration is not uniformly associated with worse outcomes in more recent cohorts, treated with anti-TNF, whereas ileal ulceration is less likely to respond to anti-TNF.^81,82^

Beyond disease location and behavior at diagnosis (e.g., penetrating disease, perianal disease), previous attempts to include complex biomarkers in personalizing CD therapy, have not been widely adopted in pediatric CD, for example, NOD2/CARD15 genetics, anti-microbial serology.^83^ According to the Beaugerie criteria, pediatric age at diagnosis alone is associated with worse disease course.^84^ Atia et al.^85–87^ have illustrated the lack of reliable biomarkers of severity in pediatric CD with available rarely RCTs able to reflect the breadth of phenotypes of the patient population in CD. Ricciuto et al.^88^ have shown the relationship between complicated disease and growth impairment with diagnostic delay. In our cohort, most patients were included shortly after diagnosis.

In the absence of widely applicable clinical, genetic, or serological biomarkers, the failure of the 17-gene transcriptional biomarker in the PROFILE study of anti-TNF use is particularly noteworthy.^89^ Derived from a CD8 T-cell transcription profile associated with the progression of disease (evidenced by a need for immunomodulatory therapy or surgery), external validation prior to the use in the PROFILE study still did not lead to added benefit in the first biomarker-stratified trial design.^90^ When we consider the complex polygenic host–microbiome interaction associated with disease development and disease course, as discussed above, markers such as tryptophan-metabolites (alone or in the future perhaps in combination with other metabotype measures), which inherently merge host genetics, immunology, and microbiome, may bridge this gap from host-focused biomarker development to an integrative disease marker. The findings in our much smaller cohort of nutritional therapy will obviously require validation in larger cohorts of similar disease phenotype and duration.

Further research into such markers may inform strategies to improve the therapeutic ceiling encountered for all drugs to date. Aden et al.^91^ have previously described that the long-term response to anti-TNF is associated with microbiome features at baseline (e.g., SCFA generating capability as a marker of a less dysbiotic state). Previous attempts to reduce lifestyle and dietary management to adding or removing a single food item have been scarce and mostly underwhelming.^49,50,92^ Recent data on the complexity of, for example, fiber supplementation, with its effect depending on the microbiome/inflammatory state of the intestine, are illustrative of the complexity required of dietary intervention, both in composition and in time.^93^ The potential of a baseline marker such as tryptophan metabolites, which does not require complex bioinformatics and is yet able to reflect the degree of dysbiosis sufficiently to predict the likelihood of diet-induced remission, is highly promising to prioritize for further studies. The link of tryptophan metabolism with several IBD-associated mechanisms through the Aryl hydrocarbon receptor (bridging microbiome, appropriate regulation of immunity warrants further translational studies given the complexity of AhR biology).^94–98^

The samples were from a previously conducted international RCT with a homogeneous and well-characterized cohort that included extensive multi-omics data. Limitations of our study include the small sample size, particularly the small number of patients not achieving remission. Previous analyses on a larger subgroup of this cohort (due to the greater availability of fecal samples at that time, before other multi-omics analyses were reported) triggered the in-depth analysis of tryptophan metabolism reported herein.

In this study, remission at Week 6 was determined solely based on the PDCAI score, in keeping with the outcomes used in the RCT. We have previously shown normalization of CRP for most children in this cohort with dietary therapy.^29^ Fecal calprotectin was markedly elevated in both groups ranging between 50 and 17,000 μg/g at entry. Calprotectin dropped significantly for both groups between Week 0 and Week 6 (Supplemental Figure 1). There was no statistically significant difference in median delta calprotectin between CDED + PEN and EEN, which represents a 49.0% and 56% drop in median calprotectin, respectively, by Week 6 (Supplemental Figure 1). Future studies are suggested to have longer follow-ups and incorporate combined analyses based on PDCIA score, systemic inflammation (CRP), mucosal inflammation (calprotectin), and microbiome/metabolome signatures to provide more insights.

Since tryptophan pathways include numerous metabolites not covered in this study, this inevitably results in a lower resolution of the analysis of complex metabolic pathways. The use of ratios to highlight differences between groups increases the number of comparisons, raising the risk of overfitting and false discoveries, especially given our small sample size. While this approach is biologically plausible and aligns with previous studies, we acknowledge this limitation and emphasize that our findings are exploratory. Future research with larger cohorts and longer follow-ups is needed to validate these results.

In conclusion, we have provided evidence across different time points within a cohort from a previous RCT that tryptophan metabolites are associated with remission with dietary therapy. Additionally, we have demonstrated clear differences in kynurenine and tryptophan metabolite ratios at baseline that are associated with diet-induced remission at Week 6. We identified a potential biomarker that could help predict remission to dietary therapy, allowing for personalized treatment and preventing unnecessary dietary restrictions. Further studies are warranted to confirm these findings.

## Supplemental Material

sj-jpg-1-tag-10.1177_17562848251323004 – Supplemental material for Tryptophan metabolites profile predict remission with dietary therapy in pediatric Crohn’s disease Supplemental material, sj-jpg-1-tag-10.1177_17562848251323004 for Tryptophan metabolites profile predict remission with dietary therapy in pediatric Crohn’s disease by Rotem Sigall Boneh, Nikki van der Kruk, Eytan Wine, Charlotte M. Verburgt, Tim G. J. de Meij, Mark L�wenberg, Krisztina B. Gecse, Nicolette Wierdsma, Joep P. M. Derikx, Wouter J. de Jonge, Geert D’Haens, Mohammed Ghiboub and Johan E. Van Limbergen in Therapeutic Advances in Gastroenterology

## Declarations

**Ethics approval and consent to participate:** The study is registered as NCT01728870. The study was approved by the local ethics board at each of the participating sites. Consent was obtained from each of the participants/family as appropriate.

**Consent for publication:** Not applicable.

**Availability of data and materials:** Metagenome and metabolome data were previously made publicly available in Levine et al. 2019, Ghiboub et al. 2022, and Verburgt et al. 2023.
