# Supplementary figures and images for: Tryptophan metabolites profile predict remission with dietary therapy in pediatric Crohn’s disease
Source: Ther Adv Gastroenterol. 2025 Feb 25;18:17562848251323004. doi: 10.1177/17562848251323004 (PMC11863242; doi:10.1177/17562848251323004)

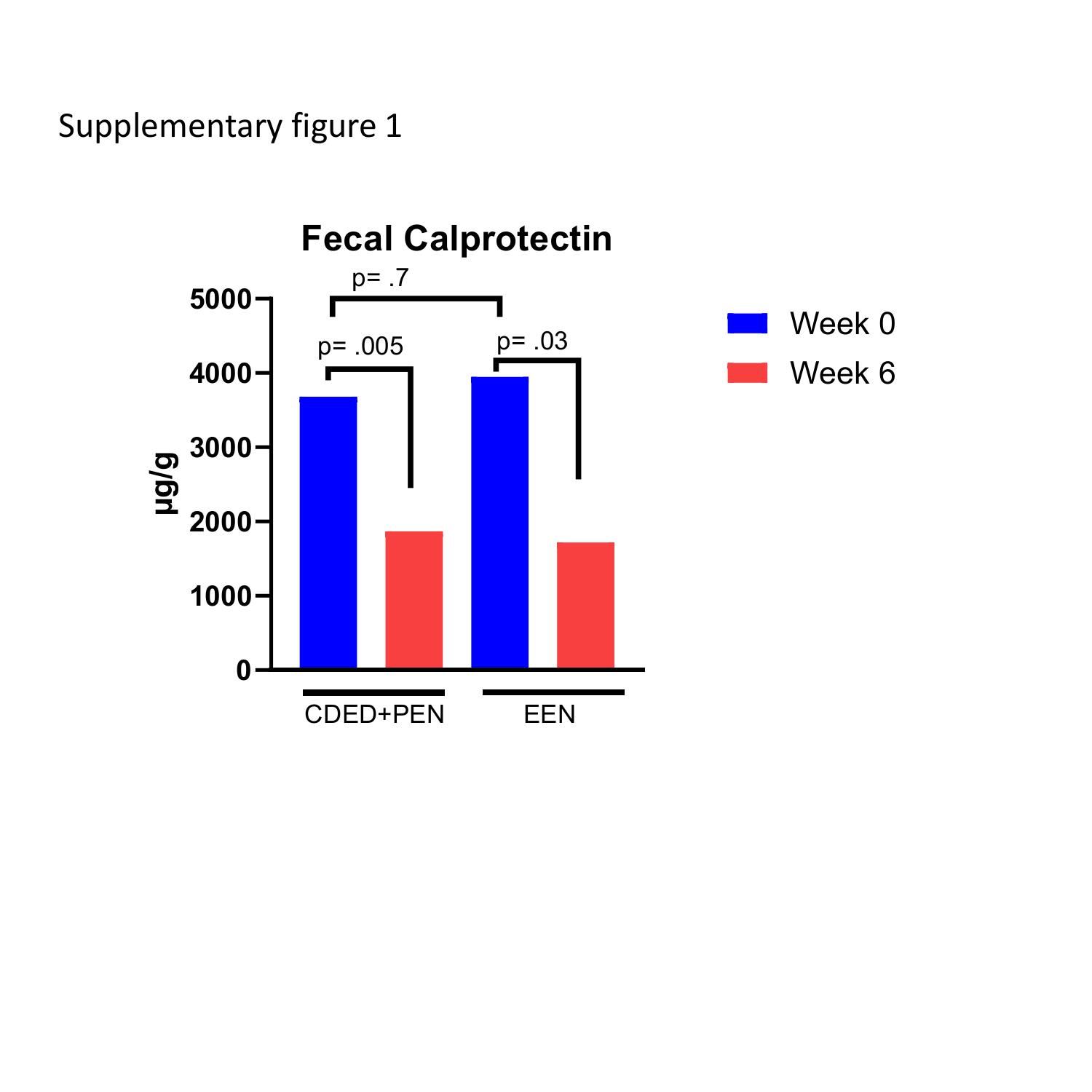

Supplement: sj-jpg-1-tag-10.1177_17562848251323004 – Supplemental material for Tryptophan metabolites profile predict remission with dietary therapy in pediatric Crohn’s disease [file sj-jpg-1-tag-10.1177_17562848251323004.jpg]
